# Host species and habitats shape the bacterial community of gut microbiota of three non-human primates: Siamangs, white-handed gibbons, and Bornean orangutans

**DOI:** 10.3389/fmicb.2022.920190

**Published:** 2022-08-16

**Authors:** Chingwen Ying, You-Shun Siao, Wun-Jing Chen, Yi-Ting Chen, Szu-Lung Chen, Yi-Lung Chen, Jih-Tay Hsu

**Affiliations:** ^1^Department of Microbiology, Soochow University, Taipei, Taiwan; ^2^Taipei Zoo, Taipei, Taiwan; ^3^Department of Animal Science and Technology, National Taiwan University, Taipei, Taiwan

**Keywords:** microbial community, non-human primates, gut microbiota, short chain fatty acids, probiotics

## Abstract

The gut microbiome is essential for a host to digest food, maintain health, and adapt to environments. Bacterial communities of gut microbiota are influenced by diverse factors including host physiology and the environment. Many non-human primates (NHPs), which are physiologically close to humans, are in danger of extinction. In this study, the community structure of the gut microbiota in three NHPs: siamangs (*Symphalangus syndactylus*, Ss), Bornean orangutans (*Pongo pygmaeus*, Pp), and white-handed gibbons (*Hylobates lar*, Hl)—housed at the largest Zoo in Taiwan were analyzed. Pp and Ss were housed in the Asian tropical rainforest area, while Hl was housed in two separate areas, the Asian tropical rainforest area and the conservation area. Bacterial community diversity of Ss, indicated by the Shannon index, was significantly higher compared with that of Hl and Pp, while the richness (Chao 1) and observed operational taxonomic units (OTUs) were similar across the three species of NHPs. Host species was the dominant factor shaping the gut microbial community structure. Beta-diversity analysis including non-metric multidimensional scaling (NMDS) and unweighted pair group method with arithmetic mean (UPGMA) suggested gut bacterial communities of Hl housed in the conservation area were closely related to each other, while the bacterial communities of Hl in the rainforest area were dispersedly positioned. Further analysis revealed significantly higher abundances of *Lactobacillus fermentum, L. murinus*, and an unclassified species of *Lactobacillus*, and a lower abundance of *Escherichia-Shigella* in Hl from the conservation area relative to the rainforest area. The ratio of *Lactobacillus* to *Escherichia-Shigella* was 489.35 and 0.013 in Hl inhabiting the conservation and rainforest areas, respectively. High abundances of *Lactobacillus* and *Bifidobacterium* and a high ratio of *Lactobacillus* to *Escherichia-Shigella* were also observed in one siamang with notable longevity of 53 years. Data from the study reveal that host species acted as the fundamental driving factor in modulating the community structure of gut microbiota, but that habitats also acted as key determinants within species. The presence and high abundance of probiotics, such as *Bifidobacterium* and *Lactobacillus*, provide potential indicators for future diet and habitat optimization for NHPs, especially in zoological settings.

## Introduction

It is well-established that mammalian gut microbiota regulates host metabolism, and that the composition of the gut microbiota dynamically changes in response to the physiological state of a host. Gut microbiota also plays key role in animal health, host physiology, and gut homeostasis (Sekirov et al., 2010; Feng et al., 2015). Moreover, studies on insects, birds, and mammals strongly suggest that inflammatory bowel disease results from dysregulated immune responses of the gut flora (Koboziev et al., 2014; Singh et al., 2017). Diversity and composition of gut microbiota are influenced by intrinsic factors, including host species, age, and sex, as well as extrinsic factors, such as lifestyle, environment, behavior, and diet.

Discovery and identification of the diverse processes orchestrating gut microbial community structure have received considerable attention, and the results of such studies are often controversial. Genetics was implicated as a major factor in gut microbial community structure, especially maternal gut microbiota on the gut microbial composition of offspring (Liu et al., 2015; Moeller et al., 2018). Host species has been identified as the dominant influential factor of gut microbial communities in various animals (Amato et al., 2019; Knowles et al., 2019). Gut microbial communities were shown to cluster according to the host species (Groussin et al., 2017) and the phenomenon phylosymbiosis (Lim and Bordenstein, 2020) was observed, whereby relationships among gut microbiomes were closely related to the phylogenetic tree of their mammalian hosts.

Other reports suggested that diet is the cardinal factor in determining the microbiome composition of the gut microbiota (Ley et al., 2008; Amato et al., 2015; Campbell et al., 2020). Accumulated data indicated that diet and habitats are the primary shaping force of the gut microbiome, with habitats having a greater impact than host species on the gut microbiota (Scott et al., 2013; Wolter et al., 2021). Narat et al. (2020) reported that the microbiota of zoo gorillas more closely resembled that of zoo chimpanzees rather than Cameroonian gorillas, suggesting the influence of diet and zoo living conditions on gut microbiota. The gut microbiomes of chimpanzees and gorillas living in US zoos were most similar to those of people who ate non-Western diets than to those of wild chimpanzees and gorillas living in a National Park (Campbell et al., 2020). The authors speculated that this might be attributed to human contact and the diet provided by the zoo.

Small-molecule metabolites of gut bacteria have received increasing attention for their potential functions in maintaining host health and digestion homeostasis. Gut bacteria can ferment dietary fibers, resistant starch, and phytonutrients in the intestine, producing short-chain fatty acids (SCFAs) that mainly comprise acetic acids, propionic acids, and butyric acids (Rechkemmer et al., 1988). SCFAs provide 5–10% of energy sources for the colonic epithelium and the gut microorganisms. The composition and abundance of SCFAs are responsive to dietary factors, such as polyphenols, dietary fibers, and fruit supplementations, as well as the gut environment of the host. Subsequently, SCFAs can influence host physiology (Morrison and Preston, 2016). In particular, butyric acid is associated with protective roles in inflammatory bowel diseases and other dysfunctions of hosts (Tang et al., 2020).

Culture-dependent and culture-independent methods are two approaches that were routinely used to analyze microbial composition. With the development of next-generation sequencing (NGS) tools, the gut microbial composition could be studied *via* 16S rRNA gene sequencing. This approach decreases the hard-to-avoid biased growth selection of the culture approach. Identification of gut microbiota and the predicted functions of the communities encoded in the microbiomes can bring new perspectives on the hosts.

In this study, PacBio third-generation sequencing was employed to sequence full-length 16S ribosomal RNA genes and explore the impacts of host species, genetics, and habitats on the diversity and composition of gut microbiota of three species of non-human primates (NHPs): *Hylobates lar, Pongo pygmaeus*, and *Symphalangus syndactylus*, abbreviated herein as Hl, Pp, and Ss, respectively. The fecal SCFA composition of the three primates was also determined. Moreover, the abundance of probiotics and potential pathogens present in the gut microbiota of white-handed gibbons inhabiting different areas of the zoo were analyzed. For animals housed in the same habitat with similar diet and management, the gut microbial community structure was distinct among the three NHP species in the study. However, the gut microbiome composition of white-handed gibbons (Hl) was influenced by their habitats, named Asian rainforest area and conservation area. The findings from the study suggest the fundamental impact of host species on the gut microbial community structure and recognize the influence of habitats on the gut microbiome. Furthermore, probiotics identified in this study provide potential indicators for more suitable diet and habitat designs for NHPs in captivity conditions, such as zoo settings.

## Materials and methods

### Animal fecal sample collection

Fecal samples of 16 NHPs (Table 1) were collected in May 2021. Gibbons are Asian apes that inhabit the rainforests of South and Southeast Asia. Ss are the largest gibbons, while Hl are among the fastest of all primates. Both have been listed as endangered primate species in the International Union for Conservation of Nature (IUCN) Red List. Pp is the only great ape that inhabits Asia and is listed as critically endangered. All primates were housed at Taipei Zoo (24.59 N, 121.35 E), the largest and most advanced zoo in Taiwan. Pp (*n* = 6) and Ss (*n* = 5) were housed in the Asian tropical rainforest area, while Hl (*n* = 6) were housed in two separate areas, the Asian tropical rainforest area and the conservation area (Table 1). The husbandry manuals for Ss (Riley, 2008), Hl (Miller, 2010), and Pp (AZA Ape Taxon Advisory Group., 2017), respectively, were used as references for housing, handling, diet, feeding routine, and management of animals. In the Asian tropical rainforest area, animals were routinely exposed to potential human contact twice daily at feeding. In the conservation area, the manger was placed outside of the enclosure with easy access for the animal, and human exposure and handling were limited. The floor of the conservation area where each animal was housed was finished with tiles and cement, while the ground of the Asian rainforest area was predominantly covered with soil with plants and shrubs. The dietary composition (percentage of crude protein, crude fat, and crude fiber) for each species is indicated in Table 1. At the time of fecal sample collection, all animals were free of symptoms of disease, and had not been on any medications for at least 3 weeks prior, except for Pp2 who has periodically received *Lactobacillus paracasei* provided by veterinary staff for his soft stool problem. Fresh fecal samples (*n* = 16) were collected in the morning and stored at −80°C before DNA extraction and SCFA extraction; one fecal sample was collected for each animal. Husbandry and experimental procedures were reviewed and approved by the Animal Use and Care Committee of Taipei Zoo.

**Table 1:** Animal subjects in the study.

**Subject**	**Species**	**Sex**	**Age**	**Habitats**	**Female parent**	**Male parent**	Diet^**^,%		
							**A**	**B**	**C**
Hl 1^*^	*Hylobates lar*	Female	32	Asian Tropical Rainforest Area			15.82^a^ (0.74)	2.50^a^ (0.01)	10.48^a^ (0.04)
Hl 2^*^^,^^#^		Male	26	Asian Tropical Rainforest Area	◦	□			
Hl 3		Male	21	Asian Tropical Rainforest Area	Hl1				
Hl 5^#^		Female	13	Conservation Area	◦	□	19.67^b,c^ (0.163)	6.28^b^ (0.48)	8.34^a^ (1.66)
Hl 6		Male	13	Conservation Area					
Hl 11		Male	20	Conservation Area					
Pp1	*Pongo pygmaeus*	Female	36	Asian Tropical Rainforest Area			19.27^c^ (0.26)	4.51^c^ (0.13)	13.50^b^ (0.69)
Pp2		Male	26	Asian Tropical Rainforest Area					
Pp3		Female	21	Asian Tropical Rainforest Area					
Pp4		Female	13	Asian Tropical Rainforest Area	Pp1				
Pp5		Female	8	Asian Tropical Rainforest Area	Pp3	Pp2			
Ss 1	*Symphalangus syndactylus*	Male	53	Asian Tropical Rainforest Area			19.75^a,b,c^ (1.37)	3.34^a,b,c^ (1.91)	20.93^a,b^ (7.34)
Ss 2^⋆^		Female	16	Asian Tropical Rainforest Area	•	Ss1			
Ss 3^⋆^		Male	14	Asian Tropical Rainforest Area	•	Ss1			
Ss 4^⋆^		Female	12	Asian Tropical Rainforest Area	•	Ss1			
Ss 5		Male	11	Asian Tropical Rainforest Area					

* Group-housed.

# Offspring of same pair of parents.

⋆ Offspring of same pair of parents.

** The data are presented as the mean (SD). A, crude protein; B, crude fat; C, crude fiber. Values within a column with different superscripts differ significantly at P < 0.001.

◦, □, • each represents an individual animal.

### DNA extraction, quantification, and quantitative PCR (qPCR)

Genomic DNA extraction was performed as previously described (Ying et al., 2018) with modification. A DNeasy PowerSoil Kit (Qiagen, Germany) was used to extract the fecal DNA according to the manufacturer's recommendations. DNA was quantified using a micro-volume nucleic acid spectrophotometer (ASP-2680, ACTGene, USA) and stored at −30°C for further analysis.

To quantify fecal bacterial abundance, qPCR with primers for the 16S rRNA gene (967F: 5′-CAACGCGAAGAACCTTACC-3′; 1046R: 5′-CGACAGCCATGCANCACCT-3′) was used as previously reported (Ying et al., 2020). A cloned fragment of the 16S rRNA gene from *Escherichia coli* served as a standard (Shiung et al., 2018). The PCR was executed in the StepOne™ Real-Time PCR System (Applied Biosystems, Singapore) with an initial denaturation at 95°C for 10 min, 40 cycles of 15 s at 95°C, 15 s at 61°C, and 1 min at 72°C. The PCR mix contained 5 μL SYBR green master mix (Applied Biosystems), 0.5 μL of each primer, and 2 μL template DNA (diluted to 0.25–1 ng/μL) in a total reaction volume of 20 μL.

### PCR-based 16S rRNA gene amplification and high-throughput sequencing

Full-length 16S rRNA genes for the PacBio library construction and sequencing were amplified by using primer set 27F (5′-AGRGTTYGATYMTGGCTCAG-3′) and 1492R (5′-RGYTACCTTGTTACGACTT-3′) to enable longer reads and thus more precise taxonomic identification. The resulting PCR products were purified *via* binding to the pre-washed AMPure PB beads (Part Number PB100-265-900). After end-repair of the products, blunt adapters were ligated, followed by exonuclease incubation to remove all un-ligated adapters and DNA. The final “SMRT bells” were annealed with primers and bound to the proprietary polymerase using the Sequel Binding Kit 2.1 (Part Number PB 101-429-300) to form the “Binding Complex.” After dilution, the library was loaded onto the instrument with Sequel Sequencing Kit 2.1 (4 rxn) (Part Number PB 101-310-500) and a Sequel™ SMRT^®^ Cell 1M v2 Tray (4 cells) (Part Number PB101-008-000) for sequencing.

### Data sequencing and processing

All raw data (^*^.fastq) were analyzed by FastQC (v0.11.5) followed by MultiQC (v0.9). Operational Taxonomic Units (OTUs) were selected using mothur v.1.39.5 (Schloss et al., 2009) with 97% identity. Chimera sequences were identified using UCHIME v4.2 (with reference data: Gold database) and replication was recognized using DADA2 (Callahan et al., 2016). Reads were filtered to screen for and remove contigs containing ambiguities. All taxa of the community were recovered at the species level and sequencing depth was sufficient according to rarefaction. The main tool of the workflow was QIIME, which contained multiple analysis process programs. Venn diagram and rarefaction curve analysis were performed using the QIIME2 software (Bolyen et al., 2019), and indexes for alpha-diversity, including observed species, Chao1, Shannon, and Simpson, were individually calculated with QIIME2. To assess bacterial community diversity across samples, beta-diversity calculations were analyzed by the principal coordinates analysis (PCoA), unweighted pair group method with arithmetic mean (UPGMA), and non-metric multidimensional scaling (NMDS) was calculated by QIIME2. Data were visualized using the R package (version 3.5.2). Linear discriminant analysis (LDA) coupled with effect size (LEfSe) was applied to evaluate differentially abundant taxa (Segata et al., 2011). Tax4Fun, an open-source R package that implements the MoP-Pro approach for whole metagenome shotgun sequencing data (Aßhauer et al., 2015), was used to predict the functional abundances of the bacterial communities. Quality-filtered sequencing data was deposited in the NCBI SRA database under BioProject ID PRJNA824704.

### Fecal SCFA extraction and analysis

Extraction and analysis of SCFAs were performed according to a previous report (Primec et al., 2017) with some modifications. Briefly, 5 g fecal samples were mixed with 15 mL double-distilled water and centrifuged. Supernatants were collected and mixed with 25% metaphosphoric acid at a ratio of 4:1 (v/v). After incubation for 30 min, samples were centrifuged at 12,000 *g* for 30 min, then supernatants were collected and subjected to sequential 0.45-μm and 0.22-μm membrane filtration. For analysis, a gas chromatograph (Clarus 500, PerkinElmer instruments, MA, USA) with Nukol™ Capillary GC Column (size × I.D. 30 m × 0.25 mm, df 0.25 μm) was used, with helium at 1.19 mL/min as the carrier gas.

### Statistical analysis

Statistical analyses were performed using R version 4.0.3 (R Core Team., 2020). Data were expressed as the mean with standard deviation and Student's *t*-test with two tails was used to determine the statistical significance of differences. The PERMANOVA [permutational multivariate analysis of variance (ANOVA)] was used to determine significant differences in beta-diversity between different groups with 999 permutations. The metrics of weighted and unweighted UniFrac distances at the OTU level were used to construct PCoA.

## Results

### Abundance of fecal bacteria of NHPs

**Figure 1:**
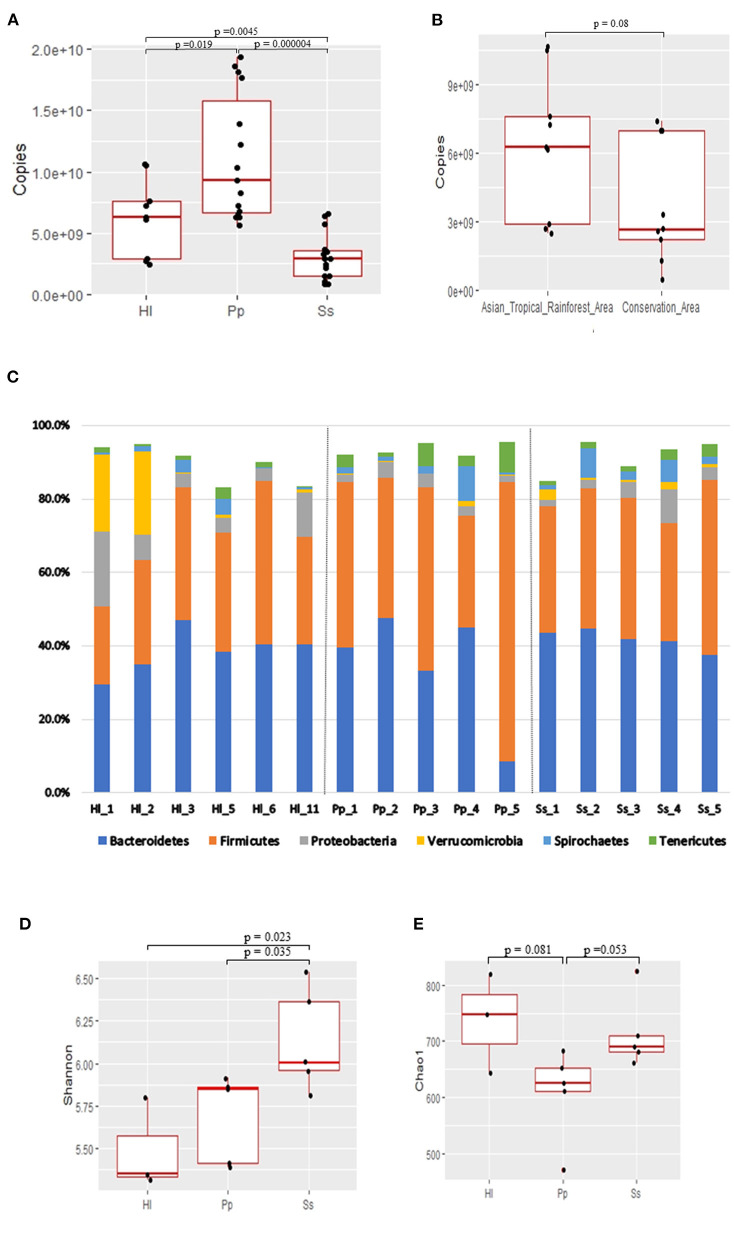
Abundance, composition, and alpha-diversity of fecal microbiomes of white-handed gibbons (Hl), Bornean orangutans (Pp), and siamangs (Ss). **(A)** Copies of the 16S rRNA gene per gram of feces were determined for the cohorts housed in the Asian tropical rainforest area. Hl, *n* = 3; Pp, *n* = 5; and Ss, *n* = 5. **(B)** The abundance of fecal bacteria in Hl inhabits either the Asian tropical rainforest area (*n* = 3) or the conservation area (*n* = 3). **(C)** Microbial community composition at the phylum taxa level. The top six dominant phyla are indicated in all samples. Alpha-diversity analysis, by **(D)** Shannon and **(E)** Chao1 indexes, of the fecal bacterial community structure of cohorts housed in the Asian tropical rainforest area. Hl, *n* = 3; Pp, *n* = 5; and Ss, *n* = 5.

The number of copies of bacterial 16S rRNA genes in feces of the NHPs was determined *via* qPCR with bacteria-specific primers, and significant differences among species were observed (Figure 1). Bornean orangutans (Pp) exhibited the highest abundance of fecal bacterial 16S rRNA genes and the Siamang (Ss) exhibited the lowest (Figure 1A). The copy number of fecal 16S rRNA genes of white-handed gibbons (Hl) resident in the Asian tropical rainforest area was 1.7-fold that of the Hl resident in the conservation area (Figure 1B).

### Microbial composition and community structure of fecal bacteria

Genomic DNA of fecal microorganisms of 16 NHPs was prepared and subjected to full-length 16S rRNA gene amplicon (average length 1,467–1,506 bp) sequencing (Supplementary Table S1). A total of 810,302 clean reads were collected and 212,853 OTUs were observed (Supplementary Table S2). The GC% was in the range of 52–57% and 24 phyla were identified in each NHP species. Microbial composition analysis indicated that Firmicutes and Bacteroidetes were the two dominant phyla in all samples (Figure 1C), constituting a combined abundance of 70.9–85.9% in each sample, except for two samples of white-handed gibbons (Hl1 and Hl2). The combined abundance of these two phyla in Hl1 and Hl2 was 50.8 and 63.5%, respectively, and the third dominant phylum, Verrucomicrobia, exhibited 21 and 22.5% abundance in Hl1 and Hl2, respectively. A low abundance of archaea was detected in all samples and the predominant archaeal genus was *Methanobrevibacter*, especially in Bornean orangutans (Supplementary Figure S1).

The richness and evenness of the gut microbiota in the three NHP species were investigated. Rarefaction analysis based on the starting cohort of 16 animals showed that gene richness approached saturation in each NHP species, and the richness of the deduced OTUs was not significantly different among the three species (Supplementary Table S2 and Supplementary Figure S2). Alpha-diversity analysis indicated that evenness of the bacterial community, as measured by the Shannon index, was significantly higher (*p*<0.05) in Ss relative to Hl and Pp (Figure 1D; Supplementary Table S3), while richness, as measured by Chao1 index, was not (Figure 1E).

**Figure 2:**
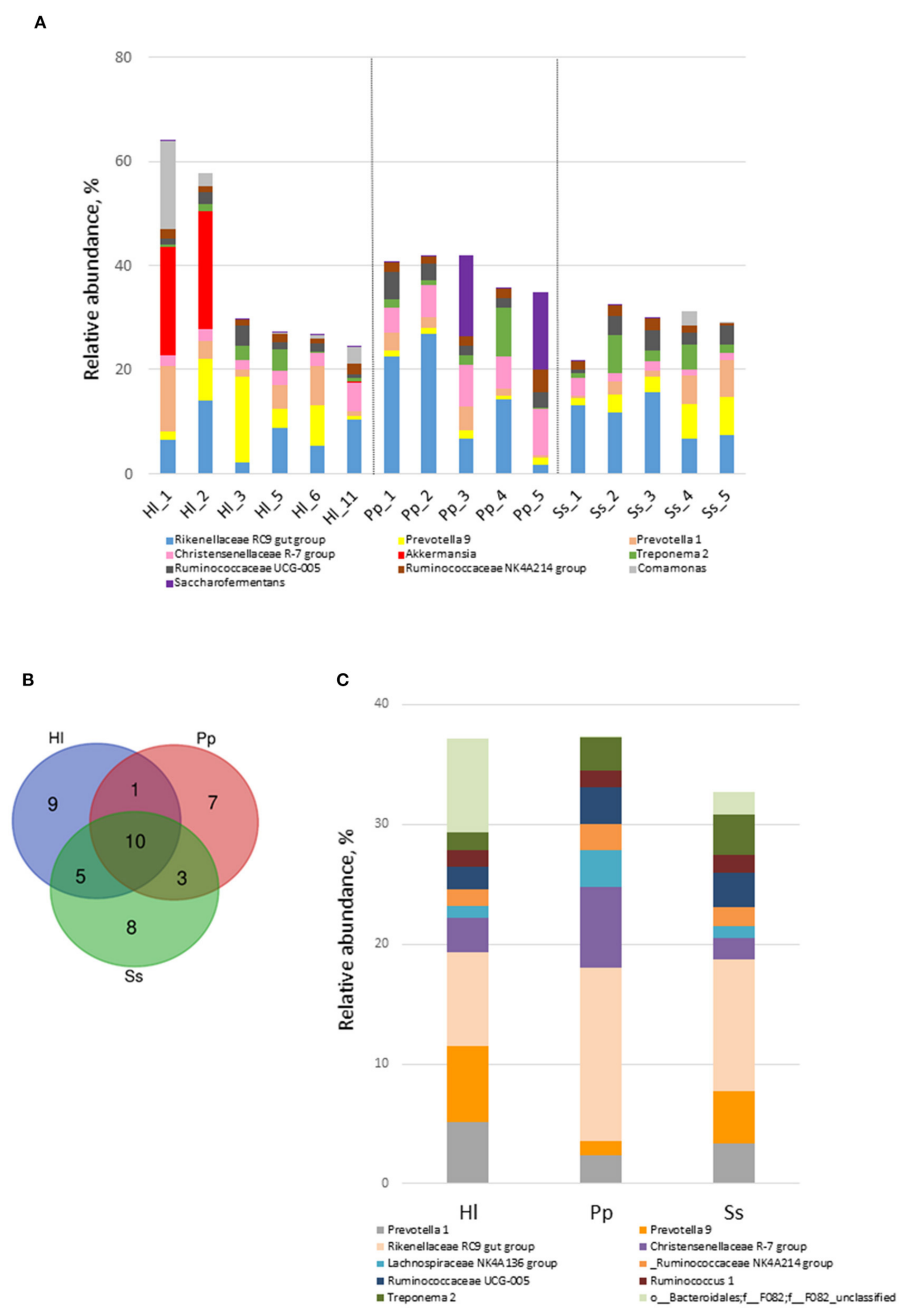
Shared genera of white-handed gibbons (Hl), Bornean orangutans (Pp), and siamangs (Ss). **(A)** The top 10 genera and their relative abundance in feces. **(B)** Venn diagram identified shared genera among the three NHP species inhabiting the Asian tropical rainforest area. Hl, *n* = 3; Pp, *n* = 5; and Ss, *n* = 5. **(C)** The 10 shared genera and their relative abundance in Hl, Pp, and Ss.

Ten dominant genera of the gut microbiota in the three NHPs were identified (Figure 2A). The top 10 genera combined represented a substantial abundance of 58–64% of the bacterial community. There was a marked abundance of the genus *Akkermansia*, which is classified in the phylum Verrucomicrobia, in the bacterial composition of Hl1 and Hl2. A Venn diagram was constructed and revealed 10 shared genera among the three NHP species (Figure 2B). Seven of the 10 shared genera were within the top 10 genera shown in Figure 2A, and it was not surprising that the combined abundance of the shared genera contributed to 37, 37, and 32.5% of the bacterial community in Hl, Pp, and Ss, respectively (Figure 2C).

**Figure 3:**
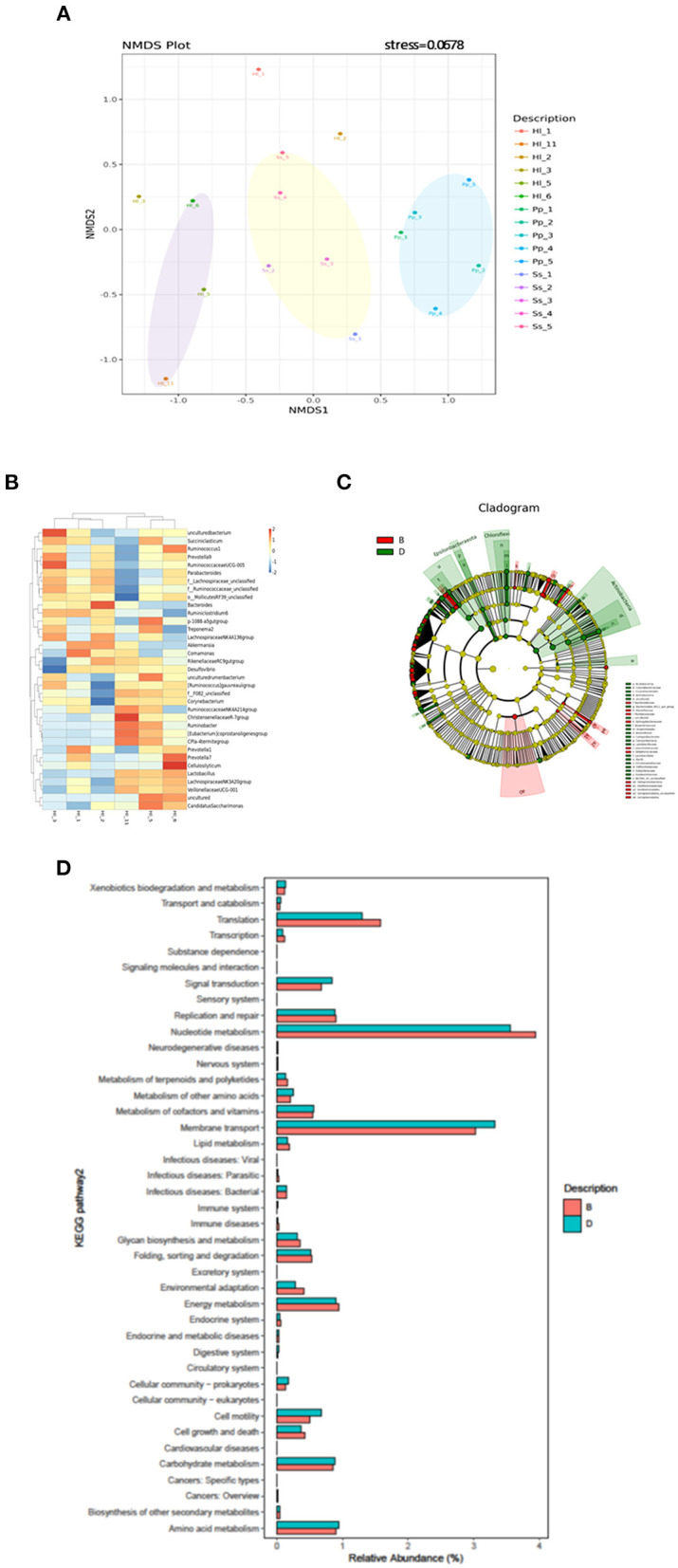
Beta-diversity analysis of fecal microbiomes of white-handed gibbons (Hl), Bornean orangutans (Pp), and siamangs (Ss). The diversity of the samples was analyzed by **(A)** non-metric multidimensional scaling (NMDS) plot of Hl (*n* = 6), Pp (*n* = 5), and Ss (*n* = 5). **(B)** Heatmap analysis at the genus taxa level. The top 35 genera of Hl housed in the rainforest area (Hl1, Hl2, and Hl3) and the conservation area (Hl5, Hl6, and Hl11) were compared. **(C)** LEfSe analysis for identification of index taxa between Hl inhabiting the rainforest area (*n* = 3) and those in the conservation area (*n* = 3). **(D)** Tax4Fun prediction of KEGG metabolic pathways of the gut microbiota in Hl inhabiting the Asian tropical rainforest area or the conservation area (*n* = 3 for each area). **(B)** Asian tropical rainforest area; **(D)** conservation area.

Beta-diversity analysis indicated that the gut microbiome of all 16 samples arranged in three clusters—Ss, Pp, and Hl were in the conservation area (Hl5, Hl6, Hl11)—in the NMDS plot (Figure 3A) with a stress value of 0.0678. The gut microbiomes of white-handed gibbons inhabiting the Asian tropical rainforest area (Hl1, Hl2, and Hl3) were sparsely positioned and showed a distant relationship to each other and the grouping of Hl5, Hl6, and Hl11. The PCoA plot confirmed this observation, showing that Hl1, Hl2, and Hl3 were distant from each other (Supplementary Figure S3A). Analysis of similarities (ANOSIM) with unweighted Unifrac revealed that the three clusters of Pp, Ss, and Hl housed in the Asian rainforest area were significantly different (*p* = 0.001, *R* = 0.685). UPGMA was used to construct agglomerative hierarchical clustering (Supplementary Figure S3B) and revealed a closer relationship of Hl2 to Hl1 than to Hl5, even though Hl2 (resident in rainforest area) and Hl5 (resident in conservation area) are offspring of the same parents. Hl1 and Hl2 both resided in the Asian tropical rainforest area. It was also noted that the linkage hierarchical clustering of Hl1 was closer to Hl2 relative to Hl3, the offspring of Hl1.

### Discriminative keystone taxa between white-handed gibbons of different habitats

To further examine how habitats might have influenced gut microbial composition, the gut microbiota of white-handed gibbons who inhabited the Asian tropical rainforest area (Hl1, Hl2, and Hl3) or the conservation area (Hl5, Hl6, and Hl11) were analyzed. The resulting heatmap demonstrated that microbial composition was associated with the habitats (Figure 3B). White-handed gibbons that inhabited the conservation area had a higher relative abundance of *Lactobacillus* and lower abundances of Bacteroides and *Ruminiclostridium* 6 compared with the gibbons inhabiting the rainforest area. A cladogram indicated that *Anaerolineae, Actinobacteria*, and *Campylobacteria* were keystone bacterial taxa of the gut microbiota in the white-handed gibbons from the conservation area (Figure 3C), while Izimaplasmatales and Xanthomonadales were the keystone taxa in animals housed in the rainforest area.

### Functional interpretations of the bacterial communities

Tax4fun2 was used to predict the function of the 16S rRNA gene sequences to evaluate potential differences in the Kyoto Encyclopedia of Genes and Genomes (KEGG) pathways. The predicted functions of the gut microbiota showed that the 41 KEGG pathways were different among white-handed gibbons in the two studied habitats (Figure 3D). Cohorts inhabiting the rainforest area had higher scores of pathways of translation and nucleotide metabolism, while cohorts inhabiting the conservation area had higher scores of pathways involved in cell motility, membrane transport, and signal transduction.

### Abundance of potential probiotic bacteria and commensal gut bacteria

**Figure 4:**
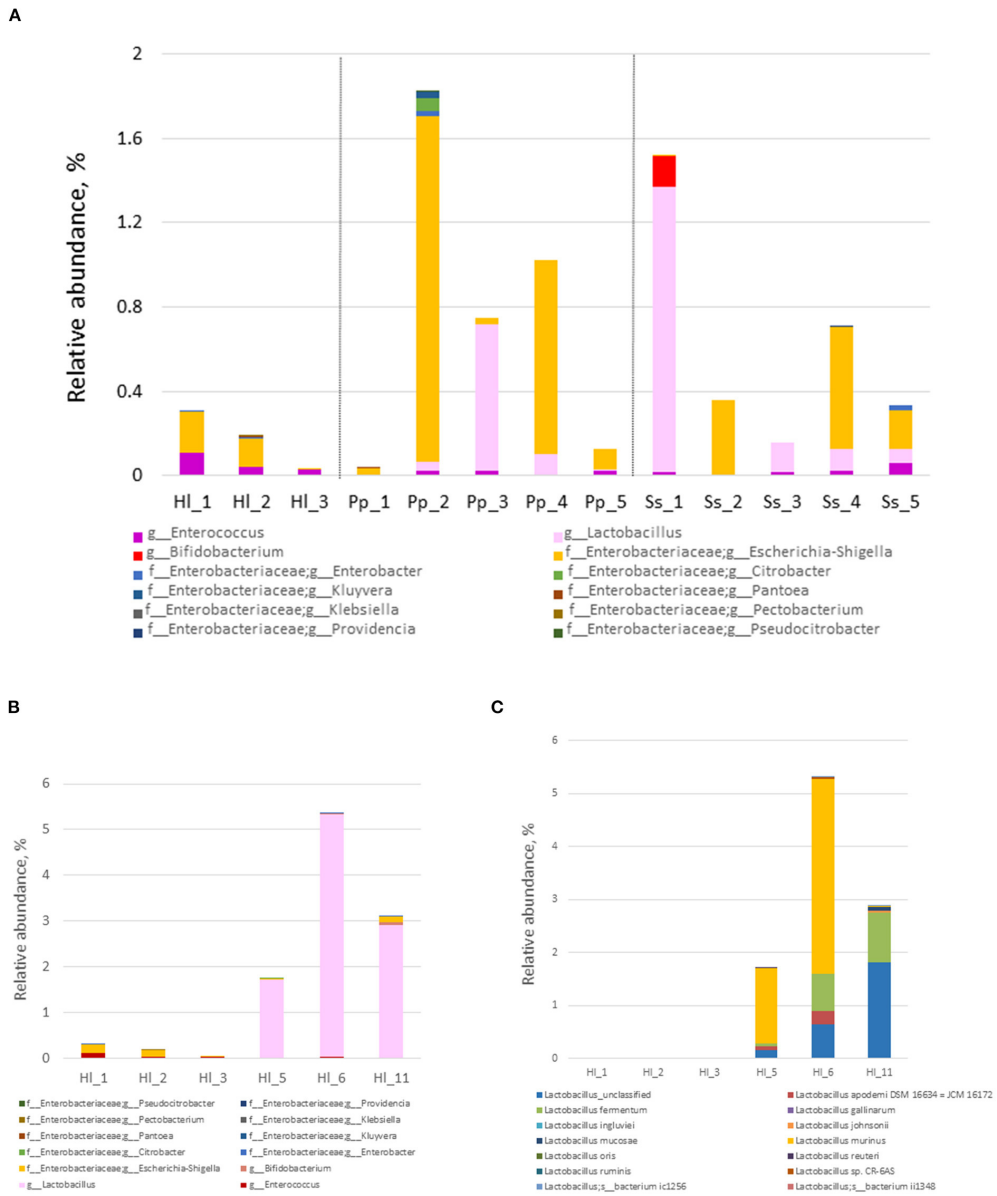
Composition and abundance of probiotics and commensal gut bacteria. **(A)** Relative abundance of commensal gut bacteria at the genus level in the fecal samples of cohorts inhabiting the rainforest area. White-handed gibbons (Hl, *n* = 3), Bornean orangutans (Pp, *n* = 5), and siamangs (Ss, *n* = 5). **(B)** Relative abundance of commensal gut bacteria at the genus level in Hl housed in the rainforest area or the conservation area. **(C)** Species of the genus *Lactobacillus* and their abundance in Hl inhabiting the rainforest area or the conservation area. Rainforest area: Hl1, Hl2, and Hl3; conservation area: Hl5, Hl6, and Hl11.

Relative abundances of potential probiotic bacteria and commensal microorganisms play a crucial role in maintaining gut homeostasis. Within the Bornean orangutans, the relative abundance of *Escherichia-Shigella* was high in Pp2 and Pp4 (Figure 4A). Among the siamangs, the relative abundances of *Lactobacillus* and *Bifidobacterium* were markedly higher in siamang Ss1, a 53-year-old male, compared with the other siamangs, including two younger males—Ss3 (age 14) and Ss5 (age 11)—who are offspring of Ss1. The combined abundance of *Lactobacillus* and *Bifidobacterium* was 1.5% in Ss1, and 0.15% and 0.07% in Ss3 and Ss5, respectively. *L. fermentum* and an unclassified species of the genus *Lactobacillus* were the two major species in the gut microbiota of siamangs.

White-handed gibbons housed in the conservation area exhibited a significantly higher abundance (*p* = 0.018) of *Lactobacillus* (3.3 ±1 .5%) compared with the white-handed gibbons inhabiting the rainforest area (0.0017 ± 0.0016%) (Figure 4B). The white-handed gibbons in the conservation area also exhibited a lower abundance of *Escherichia-Shigella* compared with the cohorts housed in the rainforest area. The ratio of the relative abundance of *Lactobacillus* over *Escherichia*-*Shigella* of white-handed gibbons inhabiting the rainforest area (0–0.02, mean = 0.010) was lower than that of the cohorts in the conservation area (22.5–1312.5, mean = 489.4). The *p-*value is 0.00235 between these two groups, computed from the Student's *t*-test, using the common logarithm of the individual value. Three species of the genus *Lactobacillus*—*L. fermentum, L. murinus*, and one unclassified species—were identified in the white-handed gibbons inhabiting the conservation area (Figure 4C).

### Composition and concentration of SCFAs in feces

**Figure 5:**
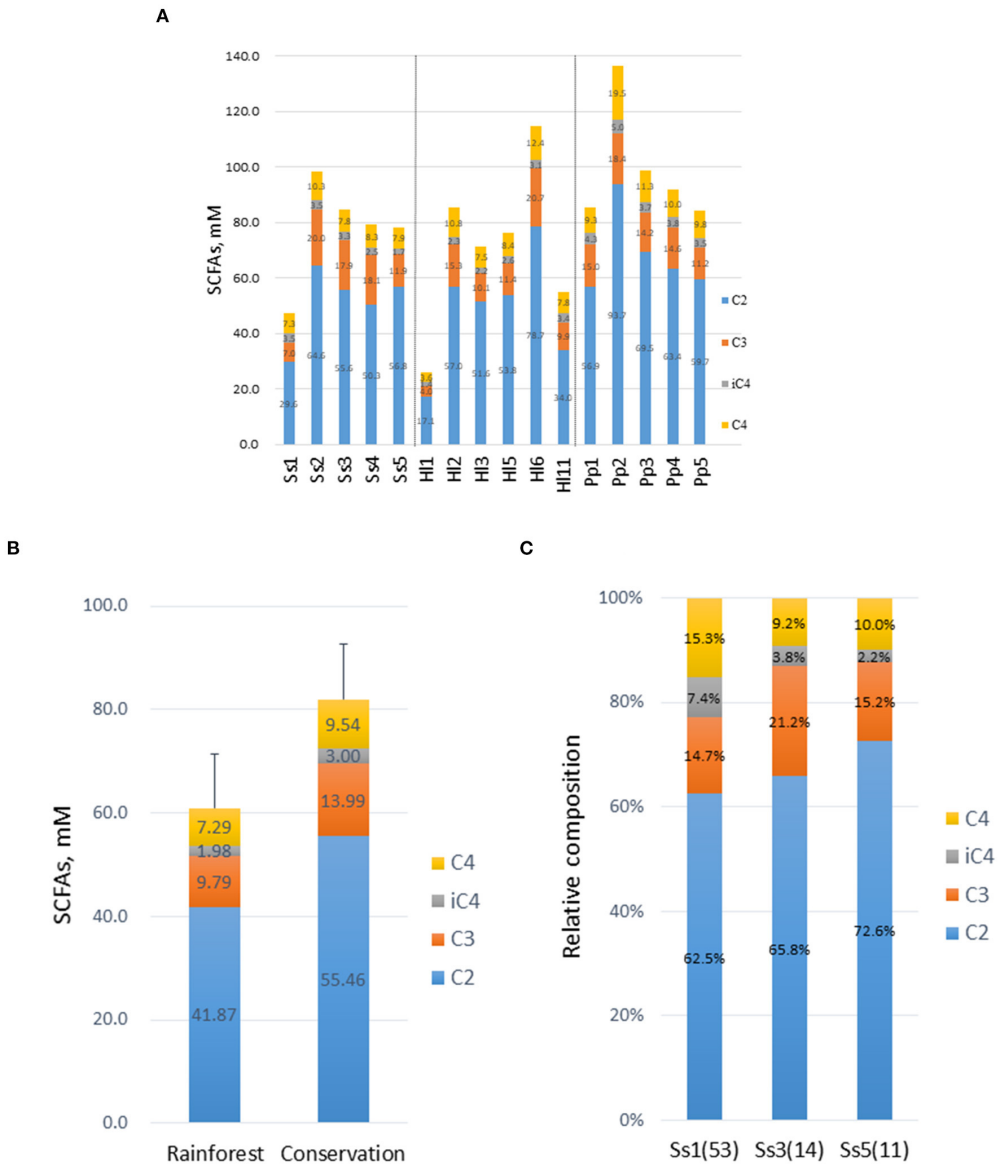
Composition and concentration of short-chain fatty acids (SCFAs) in feces. **(A)** Concentrations of SCFAs, acetic acid (C2), propionic acid (C3), isobutyric acid (iC4), and butyric acid (C4) were determined. **(B)** Concentrations of SCFAs in white-handed gibbons housed in the rainforest area (*n* = 3) or the conservation area (*n* = 3). **(C)** Composition of SCFAs in three male siamangs inhabiting the rainforest area. The age of each animal at the time of sampling is indicated in parentheses.

The concentrations of acetic acid (C2), propionic acid (C3), isobutyric acid (iC4), and butyric acid (C4) in the feces were determined (Figure 5A). All four SCFAs were detected in the feces of all 16 animals but acetic acid was most abundant, with concentrations in the range of 48.7–68.6 mM. Relative abundances of C2, C3, and C4 were similar among the three NHP species, in the range of 69–72% (C2), 15.4–20% (C3), and 11–12.6% (C4). Among the four studied SCFAs, iC4 was the only one to exhibit a significant difference (*p* < 0.05) between the Bornean orangutans (Pp) and the other two species of NHPs. The combined abundance of the four SCFAs was 1.34-fold higher in white-handed gibbons inhabiting the conservation area compared with the animals inhabiting the rainforest area (Figure 5B) with no significant changes in the composition. Siamang Ss1 showed a lower total concentration of the four SCFAs compared with the other siamangs (Figure 5A). Furthermore, the composition of the SCFAs was also different in Ss1 (Figure 5C), especially the high relative abundance of butyric acid (C4). The relative abundance of C4 in Ss1 was 1.73- and 1.61-fold higher relative to that in Ss3 and Ss5, respectively.

## Discussion

The gut microbiota played important roles in animals, including nutrient utilization and physiological functions of the host. These microorganisms are also involved in the modulation of host immunity, construction of the intestinal epithelial barrier, competitive exclusion with pathogenic bacteria by competing for nutrients, producing substances harmful to invading microbes, and affecting pH and available oxygen. In this study, the microbial compositions of three NHPs—white-handed gibbons (*Hylobates lar;* Hl), Bornean orangutans (*Pongo pygmaeus;* Pp), and siamangs (*Symphalangus syndactylus*; Ss)—were analyzed, and Firmicutes and Bacteroidetes were found to comprise most of the phyla in all three species. Yildirim et al. (2010) reported that Firmicutes occupied the largest portion (65–79%), followed by Bacteroidetes (5.5–19.3%), of the genomic sequences of a species of the NHP genus *Colobus*. Relative abundances of Firmicutes and Bacteroidetes of the NHPs in this study were 21.3–75.8% and 8.7–47.7%, respectively. This observation is more consistent with a study on cynomolgus macaques (*Macaca fascicularis*) in that the abundance of Firmicutes was either comparable to or slightly higher than that of Bacteroidetes (Nagpal et al., 2018).

Alpha-diversity analysis showed that the evenness of gut microbiota of siamangs, indicated by the Shannon index, was significantly higher compared with that of the white-handed gibbons and Bornean orangutans, but the richness, indexed by Chao 1, was indifferent among the three species of NHPs. However, the relatively small group sizes are the limitations of this study and it means that representability is a concern. Comparison with other studies on NHPs (Jia et al., 2018; Nagpal et al., 2018), including northern white-cheeked gibbons, revealed that their numbers of observed species, Chao1, and Shannon indexes, were similar to or even smaller than those observed in this study. The diversities reported in a recent study on the gut microbiota of gibbons of the genera *Hylobates* (*n*=9) and *Symphalangus* (*n*=4) housed in Shanghai Zoo in China (Lan et al., 2022) were similar to ours.

Host species appeared to be the major factor orchestrating the bacterial community structure based on beta-diversity analysis in the present study. Amato et al. (2019) and Yildirim et al. (2010) reported similar conclusions when the gut microbiome of wild NHPs was analyzed. Another study suggested habitat, not phylogeny, was the dominant control factor for gut microbial composition since the gut microbiota of zoo-living gorillas was closer to that of the chimpanzees housed in the same zoo than to that of gorillas living in Cameroon (Narat et al., 2020). Whether the target NHPs were wild or captive was considered to partially account for the discrepancy of the results in different studies (Amato et al., 2019). In this study, the impact of phylogeny on the gut bacterial community structure of the three species of NHPs housed in similar zoo settings was observed. The higher percentage of protein, fat, and fiber in the diet of Bornean orangutans (Table 1) could potentially affect the gut microbiome. However, the microbiomes of Bornean orangutans and siamangs were distributed into two clusters despite the similar composition of their diet (Figure 3; Table 1). Furthermore, siamangs and the white-handed gibbons housed in the Asian rainforest area received a similar diet and their gut microbiomes were grouped into separate clusters. This suggested that host species exhibited a greater impact than diet on gut microbial community structure.

Within the same NHP species, habitats seemed to outweigh genetics. The white-handed gibbons Hl2, resident in the rainforest area, and Hl5, housed in the conservation area, were offspring of the same parents. UPGMA displayed a closer distance of Hl2 to Hl1, which was not directly genetically related to Hl2 (Table 1; Supplementary Figure S3B). Since Hl1 and Hl2 were group-housed in the rainforest area, the environment appeared to shape the microbial community more profoundly, relative to genetics. Moeller et al. (2018) observed maternal effects on the gut microbial composition of offspring. In this study, such effects appeared to be trivial or undetectable. White-handed gibbon Hl1 is the mother of Hl3 and they did not have the closest relationship in UPGMA. Bornean orangutans Pp1 and Pp3 are the mothers of Pp4 and Pp5, respectively (Table 1). None of the mother-offspring pairs clustered closely. However, siamangs Ss2, Ss3, and Ss4, who were offspring of the same parents, showed similarities in their bacterial community, evidenced in NMDS, PCoA plot, and UPGMA (Figure 3A; Supplementary Figure S3).

The impact of habitats on the composition and diversity of the gut bacterial community of white-handed gibbons was explored further. The abundance of *Lactobacillus* increased, while the abundance of Bacteroides and *Ruminiclostridium*6 decreased in animals inhabiting the conservation area. LEfSe analysis indicated that *Anaerolineae, Actinobacteria*, and *Campylobacteria* were key bacterial genera in gibbons from the conservation area, and Izimaplasmatales and Xanthomonadales were key taxa in gibbons from the rainforest area (Figure 3C). Xia et al. (2016) identified the *Anaerolineae* as one of the core and dominant bacterial taxa in anaerobic digestive systems. The order Izimaplasmatales (phylum Tenericutes), cell-wall-less bacteria, are often parasitic or commensal to eukaryotic hosts (Skennerton et al., 2016). The order Xanthomonadales (class γ-Proteobacteria) can survive in very diverse habitats due to their flexible physiological characteristics. This order includes plant and human pathogens and non-pathogenic environmental bacteria (Saddler and Bradbury, 2005). The presence of Xanthomonadales might partially provide the elevated metabolic activity in nucleotide metabolism and translation as observed in the gibbons inhabiting the rainforest area (Figure 3D). The biological significance of these enhanced biochemical functions remains to be determined. It is conceivable that multiple factors may contribute to the influence of habitat on gut microbiomes observed in the study. Differences in the percentage of crude protein and fat in the diet (Table 1), the environment, and the frequency of potential human contact, either directly or indirectly, may cause changes in the gut bacterial composition.

The composition of probiotics and commensal bacteria in the gut microbiota of white-handed gibbons revealed a higher abundance of *Lactobacillus* and a higher ratio of *Lactobacillus* to *Escherichia-Shigella* in animals inhabiting the conservation area relative to animals in the rainforest area. At the species level, *L. fermentum, L. murinus*, and an unclassified species of the genus *Lactobacillus* were dominant in gibbons of the conservation area. The Gram-positive bacterium *L. fermentum* can enhance immunologic responses as well as prevent community-acquired gastrointestinal and upper respiratory infections (Naghmouchi et al., 2020). In addition, a higher proportion of *L. murinus* in microbiota correlated with lower necrotizing enterocolitis scores (Isani et al., 2018). A markedly low ratio of *Lactobacillus* to *Escherichia-Shigella* was observed in Bornean orangutan Pp2 (0.026), who frequently experienced soft stool problems; the average ratio for other Bornean orangutans (Pp1, Pp3–5) was 6.44. Whether this ratio can be used as an indicator of gut homeostasis requires further investigation.

The recorded maximum longevity of white-handed gibbons is 43 years in captivity (Weigl, 2005), and the lifespan in the wild is likely to be lower. Siamangs, being the largest gibbons, probably have a shorter lifespan compared with other members of the family. One of the siamangs in this study, Ss1, was 53 years old at the time of sampling and is still living. Ss1 has been noticed for his ability to maintain gut homeostasis and is seldom affected by season or diet variables. The abundances of probiotics and *Escherichia*-*Shigella* of Ss1 were compared, and the ratio of *Lactobacillus* to *Escherichia*-*Shigella* in Ss1 was 9.5- and 411.9-fold greater than that in Ss3 and Ss5, respectively. In addition to *Lactobacillus*, the presence of the genus *Bifidobacterium* was also noted in Ss1. Bifidobacteria, which commonly inhabit primate guts, are beneficial contributors to host wellbeing. These bacteria are important natural commensals and encode many genes for carbohydrate metabolism. Therefore, bifidobacteria can potentially metabolize various dietary carbohydrates in the gut (Pokusaeva et al., 2011). Although members of the genus *Bifidobacterium* can persist throughout the lifespan of primates, the abundance of *Bifidobacterium* in the gut microbiota decreases with aging (Stewart et al., 2018). Relative to adulthood, infant chimpanzee and infant human guts were both enriched in *Bifidobacterium* (Reese et al., 2021). Therefore, it is impressive that Ss1 at age 53 presented a higher abundance of *Bifidobacterium* relative to Ss3 and Ss5, aged 14 years and 11 years, respectively. Beneficial bacteria, such as *Bifidobacterium* and *Lactobacillus*, have been shown to improve the intestinal environment and yield a positive effect on metabolism, immunity, and nerve responses (Han et al., 2021). Further isolation and characterization of these probiotics from animal feces provide a promising application to improve animal welfare, which is particularly important for animals in a zoological environment.

Gut microbiota can produce small-molecule metabolites that affect the performance and health of animals. In the hindgut of NHPs, microorganisms ferment and degrade plants or convert non-digestible plant feeds into SCFAs, predominantly *via* anaerobic fermentation (Rechkemmer et al., 1988). The most abundant SCFAs are acetate (C2), propionate (C3), and butyrate (C4). The type and abundance of SCFAs produced in animal guts depend on the quantity of substrate available for microbial metabolism and the composition of the microbiota. SCFAs undergo an effective absorption from the colonic lumen and account for 10% of the daily energy intake in humans. Both acetic acid and propionic acid are mainly absorbed while butyric acid is a significant energy source for colonocytes and can modulate cell differentiation and mucus development. In addition, butyric acid can stimulate intestinal gluconeogenesis mediated through the cyclic adenosine 3′,5′-monophosphate (cAMP) signal pathway (De Vadder et al., 2014). In this study, the composition and concentrations of SCFAs were similar among the three NHP species, but a 35% increase in the total amount of SCFAs was detected in white-handed gibbons in the conservation area compared with the gibbons in the rainforest area (Figure 5B). The relative abundance of butyric acid in Ss1 was higher compared with that in Ss3 and Ss5. However, caution must be exercised in integrating these results since more than 90% of SCFAs are absorbed in the cecum and the colon *via* diffusion of protonated SCFAs and anion exchange, and only 5–10% of SCFAs are excreted in feces. Thus, the composition and abundance of fecal SCFAs might not represent the condition occurring in the gut. Nevertheless, the composition and abundance of fecal SCFAs provide a potential marker for estimating the physiology of animals and warrant further investigation.

Fecal samples were the sole source of the microbiome in this study. The gut microbial structure of individual digestive chambers cannot be fully revealed by fecal samples alone. Qi et al. (2021) reported that the alpha-diversity of ileal microbiota was lower compared with that of the colon in pigs, and different dominant genera were identified in colonic and ileal microbial communities. However, severe discomfort and suffering to the animals are unavoidable when invasive approaches are employed to collect these samples. For the endangered NHPs in this study, obtaining such samples is even more complex.

NHPs are close phylogenetic relatives and ancestors to humans and thus provide an excellent model for studying diet-microbiome interactions. NHPs, being in danger of extinction, are popular exhibitions in zoological settings. Appropriate diet and management for optimal care of these animals require an understanding of their microbial community. However, compared with clinical, rodent, and ruminant studies, research targeting the gut microbiome of NHPs is limited. In this study, the gut microbiome of three NHP species was shaped primarily by host species (Figure 3), congruent with previous findings (Yildirim et al., 2010; Amato et al., 2019). Within the same host species, habitats demonstrated influential effects on the gut bacterial community that overpowered the maternal effects on these microbiotas. The gut microbiota of white-handed gibbons housed in the conservation area comprised a higher portion of *Lactobacillus* and a higher ratio of *Lactobacillus* to *Escherichia*-*Shigella* compared with the white-handed gibbons inhabiting the rainforest area. In summary, the species of the three studied NHPs in captivity exhibited dominant effects in shaping the gut microbiome. Within the same species, habitats resulted in differences in microbial composition. Findings from the study provide fundamental information on the gut microbiota of NHPs and indications of potential probiotics for further investigation and utilization in promoting quality animal management.

## Data availability statement

The datasets presented in this study can be found in online repositories. The names of the repository/repositories and accession number(s) can be found below: https://www.ncbi.nlm.nih.gov/, bioproject/824704.

## Ethics statement

The animal study was reviewed and approved by Animal Use and Care Committee of the Taipei Zoo.

## Author contributions

CY, Y-TC, S-LC, Y-LC, and J-TH designed research. Y-SS and W-JC performed research. CY, Y-SS, W-JC, and J-TH analyzed data. CY and J-TH wrote the paper. All authors contributed to the article and approved the submitted version.

## Conflict of interest

The authors declare that the research was conducted in the absence of any commercial or financial relationships that could be construed as a potential conflict of interest.

## Publisher's note

All claims expressed in this article are solely those of the authors and do not necessarily represent those of their affiliated organizations, or those of the publisher, the editors and the reviewers. Any product that may be evaluated in this article, or claim that may be made by its manufacturer, is not guaranteed or endorsed by the publisher.

## Supplementary material

Supplementary Figure S1 The relative abundance of archaea in the gut microbiota of white-handed gibbons (Hl), Bornean orangutans (Pp), and siamangs (Ss).

Supplementary Figure S2 Rarefactions of 16 sequenced samples.

Supplementary Figure S3 Beta-diversity analysis of the fecal microbiome of white-handed gibbons, Bornean orangutans, and siamangs. The diversity of samples was analyzed by **(A)** Principal coordinates analysis (PCoA) plot. **(B)** Unweighted pair group method with arithmetic mean (UPGMA) clustering, of white-handed gibbons (Hl, *n* = 6), Bornean orangutans (Pp, *n* = 5), and siamang (Ss, *n* = 5).

Supplementary Table S1 General characteristics of sequences of each sample.

Supplementary Table S2 The number of OUT identified in each sample.

Supplementary Table S3 Alpha-diversity of each sample sequenced.
