# Supplementary material for: Host species and habitats shape the bacterial community of gut microbiota of three non-human primates: Siamangs, white-handed gibbons, and Bornean orangutans
Source: Front Microbiol. 2022 Aug 16;13:920190. doi: 10.3389/fmicb.2022.920190 (PMC9424820; doi:10.3389/fmicb.2022.920190)
Supplement: Supplementary Figure S1 — The relative abundance of archaea in the gut microbiota of white-handed gibbons (Hl), Bornean orangutans (Pp), and siamangs (Ss). [file Presentation_2.PPTX]

## Slide 1
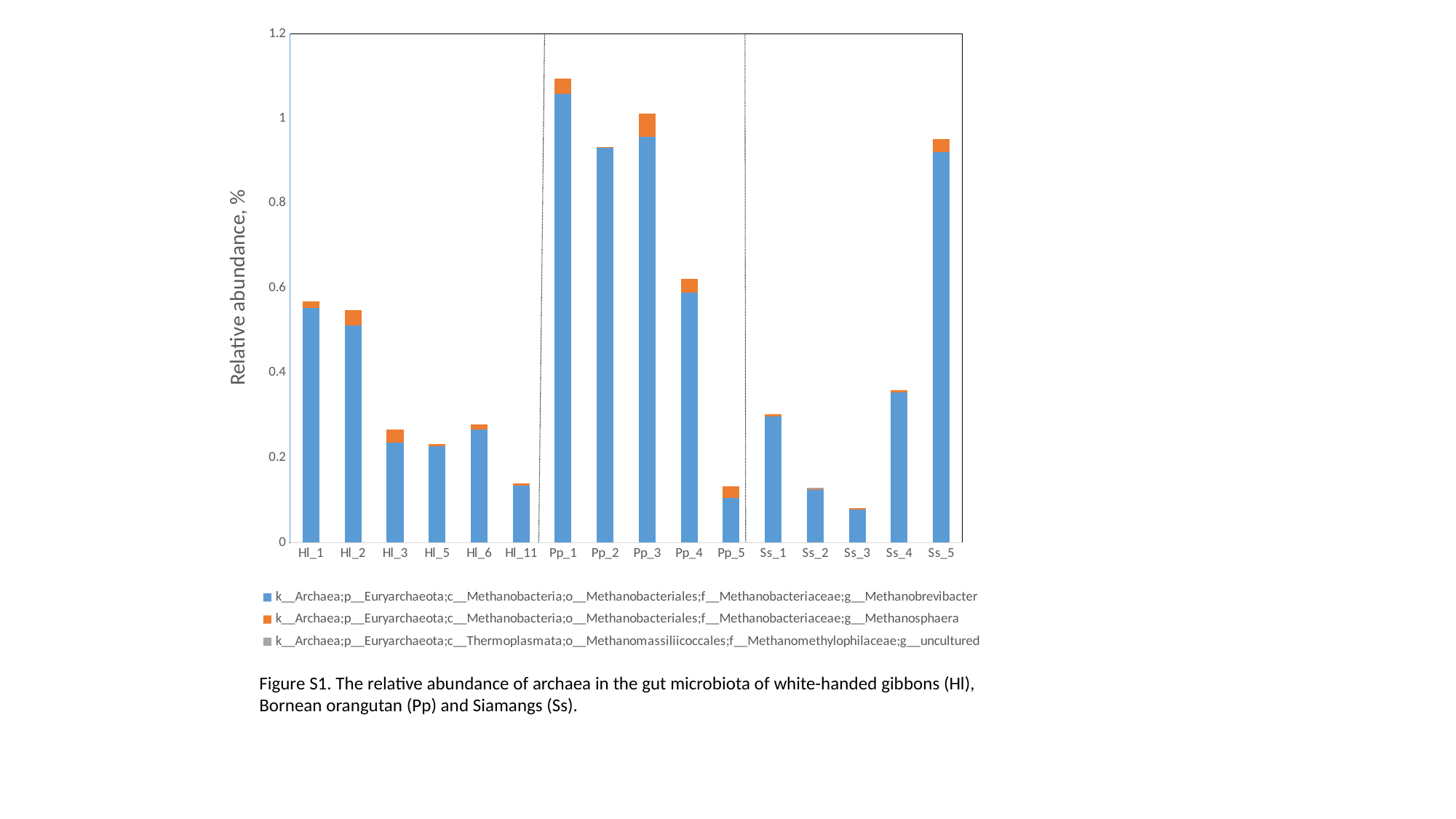

### Chart
| Category | k__Archaea;p__Euryarchaeota;c__Methanobacteria;o__Methanobacteriales;f__Methanobacteriaceae;g__Methanobrevibacter | k__Archaea;p__Euryarchaeota;c__Methanobacteria;o__Methanobacteriales;f__Methanobacteriaceae;g__Methanosphaera | k__Archaea;p__Euryarchaeota;c__Thermoplasmata;o__Methanomassiliicoccales;f__Methanomethylophilaceae;g__uncultured |
|---|---|---|---|
| Hl_1 | 0.552544004688252 | 0.0167437577178258 | 0.0 |
| Hl_2 | 0.512831160202641 | 0.0352960717548376 | 0.0 |
| Hl_3 | 0.235945453869877 | 0.0297254115111656 | 0.0 |
| Hl_5 | 0.22687966079631 | 0.00557900805236829 | 0.0 |
| Hl_6 | 0.267098340752732 | 0.0121408336705787 | 0.0 |
| Hl_11 | 0.134505361270034 | 0.00568332512408593 | 0.0 |
| Pp_1 | 1.05789294899791 | 0.0363953996214878 | 0.0 |
| Pp_2 | 0.931278821279946 | 0.00224946575188393 | 0.0 |
| Pp_3 | 0.95715135885737 | 0.0545526681214045 | 0.0 |
| Pp_4 | 0.589293836144674 | 0.0336174336055686 | 0.0 |
| Pp_5 | 0.104979688712401 | 0.0273860057510612 | 0.0 |
| Ss_1 | 0.296957519071074 | 0.00533456621085762 | 0.0 |
| Ss_2 | 0.124902205690599 | 0.00137255171088571 | 0.00274510342177142 |
| Ss_3 | 0.0771283248561661 | 0.00416909864087384 | 0.0 |
| Ss_4 | 0.354338914750226 | 0.00463188123856504 | 0.0 |
| Ss_5 | 0.920649817881165 | 0.0312842171124668 | 0.0 |Figure S1. The relative abundance of archaea in the gut microbiota of white-handed gibbons (Hl), Bornean orangutan (Pp) and Siamangs (Ss).

## Slide 2
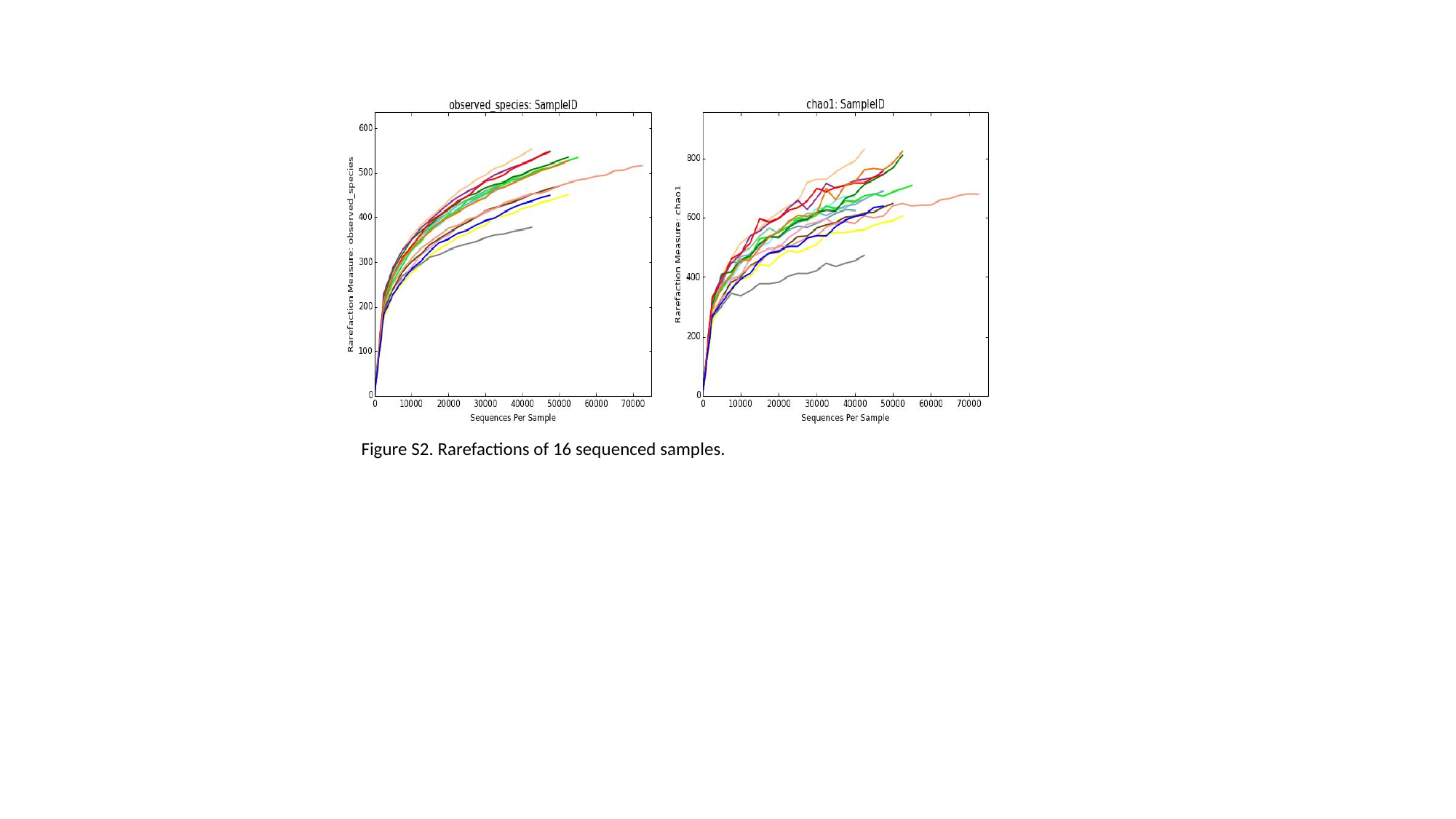

Figure S2. Rarefactions of 16 sequenced samples.

## Slide 3
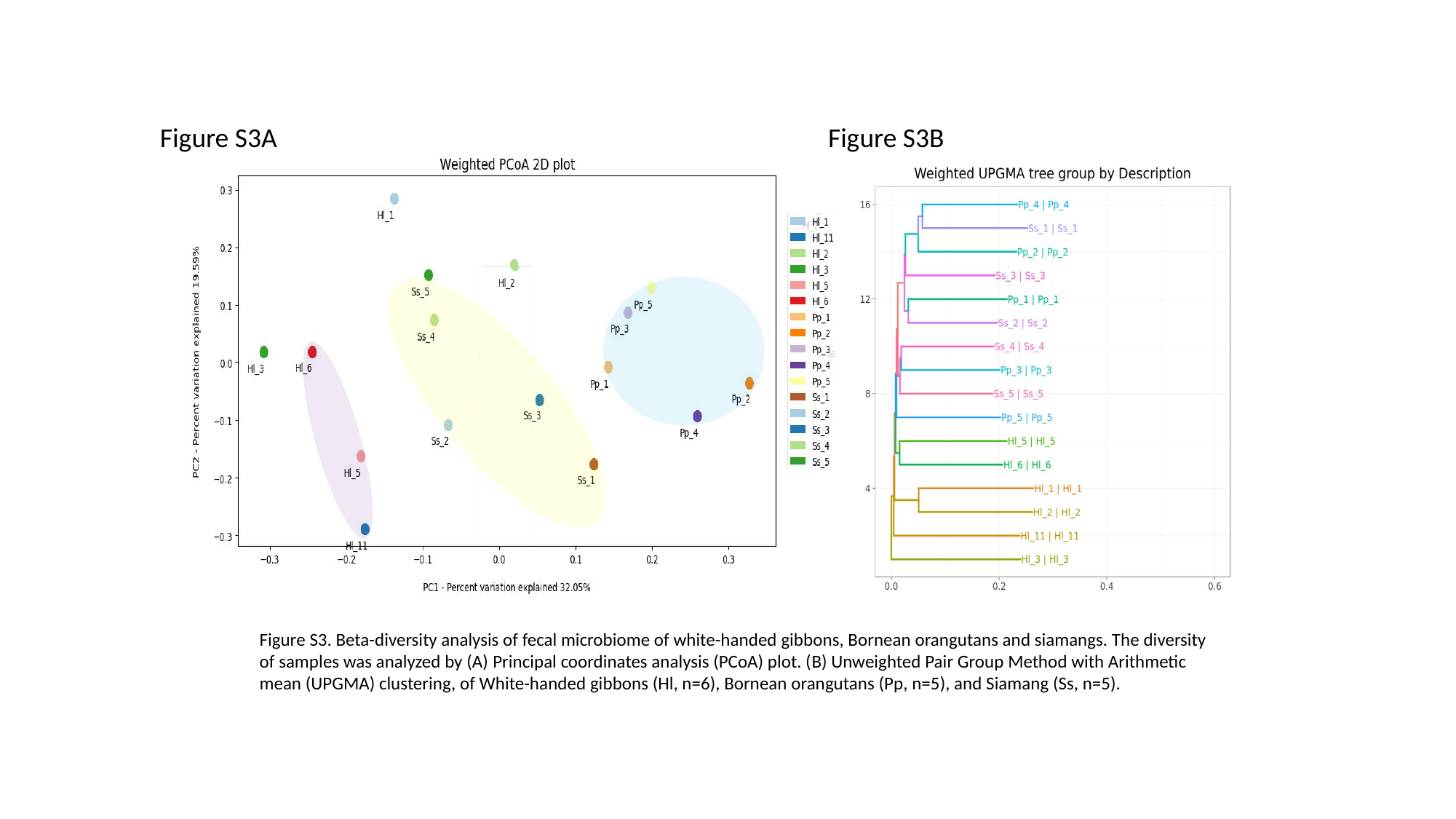

Figure S3A
Figure S3B
Figure S3. Beta-diversity analysis of fecal microbiome of white-handed gibbons, Bornean orangutans and siamangs. The diversity of samples was analyzed by (A) Principal coordinates analysis (PCoA) plot. (B) Unweighted Pair Group Method with Arithmetic mean (UPGMA) clustering, of White-handed gibbons (Hl, n=6), Bornean orangutans (Pp, n=5), and Siamang (Ss, n=5).
