# Supplementary material for: Host species and habitats shape the bacterial community of gut microbiota of three non-human primates: Siamangs, white-handed gibbons, and Bornean orangutans
Source: Front Microbiol. 2022 Aug 16;13:920190. doi: 10.3389/fmicb.2022.920190 (PMC9424820; doi:10.3389/fmicb.2022.920190)
Supplement: Supplementary Table S1 — General characteristics of sequences of each sample. [file Presentation_1.PPTX]

## Slide 1
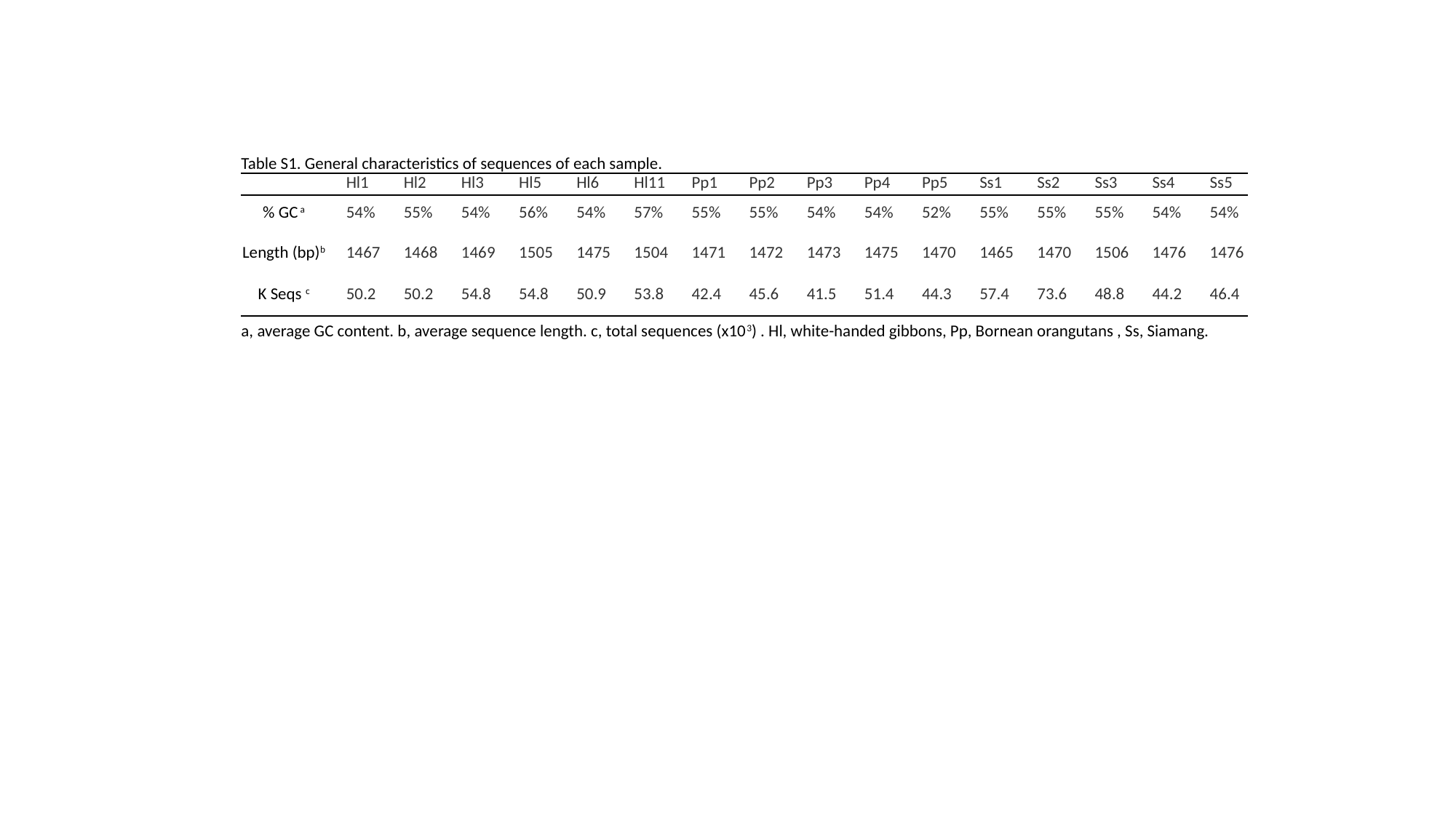

| Table S1. General characteristics of sequences of each sample. | | | | | | | | | | | | | | | | |
| --- | --- | --- | --- | --- | --- | --- | --- | --- | --- | --- | --- | --- | --- | --- | --- | --- |
| | Hl1 | Hl2 | Hl3 | Hl5 | Hl6 | Hl11 | Pp1 | Pp2 | Pp3 | Pp4 | Pp5 | Ss1 | Ss2 | Ss3 | Ss4 | Ss5 |
| % GC a | 54% | 55% | 54% | 56% | 54% | 57% | 55% | 55% | 54% | 54% | 52% | 55% | 55% | 55% | 54% | 54% |
| Length (bp)b | 1467 | 1468 | 1469 | 1505 | 1475 | 1504 | 1471 | 1472 | 1473 | 1475 | 1470 | 1465 | 1470 | 1506 | 1476 | 1476 |
| K Seqs c | 50.2 | 50.2 | 54.8 | 54.8 | 50.9 | 53.8 | 42.4 | 45.6 | 41.5 | 51.4 | 44.3 | 57.4 | 73.6 | 48.8 | 44.2 | 46.4 |
| a, average GC content. b, average sequence length. c, total sequences (x103) . Hl, white-handed gibbons, Pp, Bornean orangutans , Ss, Siamang. | | | | | | | | | | | | | | | | |

## Slide 2
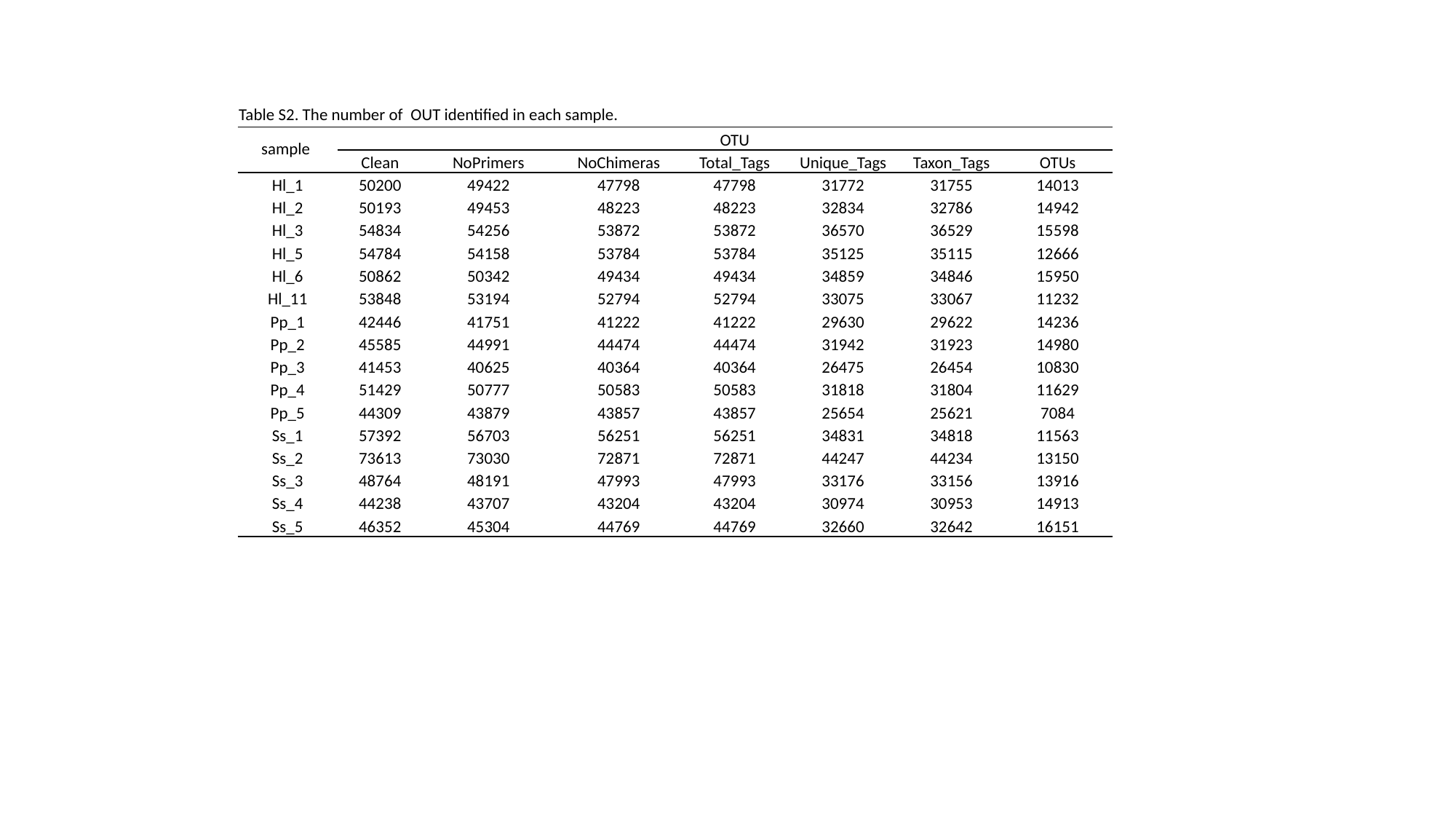

| Table S2. The number of OUT identified in each sample. | | | | | | | |
| --- | --- | --- | --- | --- | --- | --- | --- |
| sample | | | | OTU | | | |
| | Clean | NoPrimers | NoChimeras | Total\_Tags | Unique\_Tags | Taxon\_Tags | OTUs |
| Hl\_1 | 50200 | 49422 | 47798 | 47798 | 31772 | 31755 | 14013 |
| Hl\_2 | 50193 | 49453 | 48223 | 48223 | 32834 | 32786 | 14942 |
| Hl\_3 | 54834 | 54256 | 53872 | 53872 | 36570 | 36529 | 15598 |
| Hl\_5 | 54784 | 54158 | 53784 | 53784 | 35125 | 35115 | 12666 |
| Hl\_6 | 50862 | 50342 | 49434 | 49434 | 34859 | 34846 | 15950 |
| Hl\_11 | 53848 | 53194 | 52794 | 52794 | 33075 | 33067 | 11232 |
| Pp\_1 | 42446 | 41751 | 41222 | 41222 | 29630 | 29622 | 14236 |
| Pp\_2 | 45585 | 44991 | 44474 | 44474 | 31942 | 31923 | 14980 |
| Pp\_3 | 41453 | 40625 | 40364 | 40364 | 26475 | 26454 | 10830 |
| Pp\_4 | 51429 | 50777 | 50583 | 50583 | 31818 | 31804 | 11629 |
| Pp\_5 | 44309 | 43879 | 43857 | 43857 | 25654 | 25621 | 7084 |
| Ss\_1 | 57392 | 56703 | 56251 | 56251 | 34831 | 34818 | 11563 |
| Ss\_2 | 73613 | 73030 | 72871 | 72871 | 44247 | 44234 | 13150 |
| Ss\_3 | 48764 | 48191 | 47993 | 47993 | 33176 | 33156 | 13916 |
| Ss\_4 | 44238 | 43707 | 43204 | 43204 | 30974 | 30953 | 14913 |
| Ss\_5 | 46352 | 45304 | 44769 | 44769 | 32660 | 32642 | 16151 |
| | | | | | | | |

## Slide 3
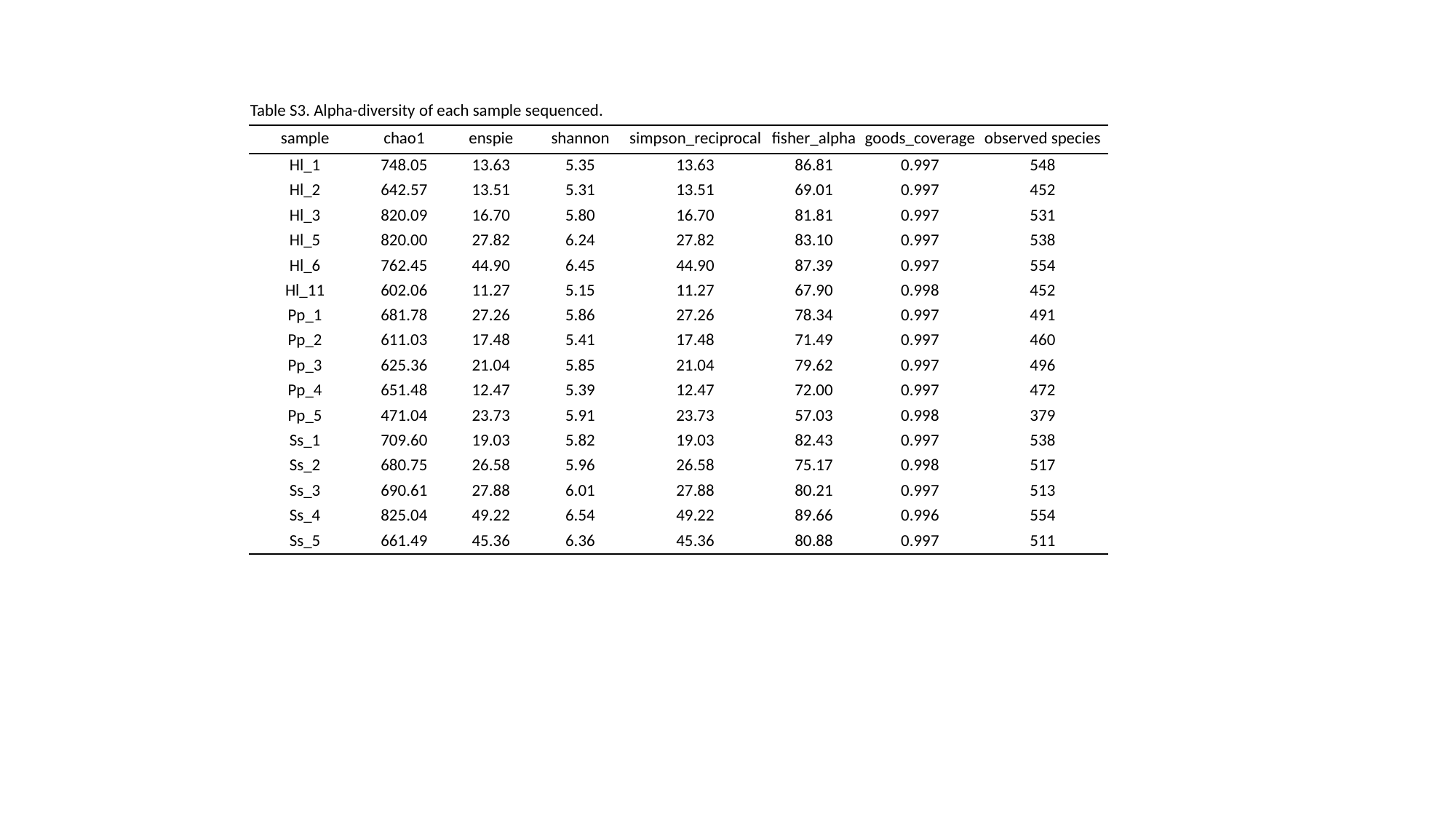

| Table S3. Alpha-diversity of each sample sequenced. | | | | | | | |
| --- | --- | --- | --- | --- | --- | --- | --- |
| sample | chao1 | enspie | shannon | simpson\_reciprocal | fisher\_alpha | goods\_coverage | observed species |
| Hl\_1 | 748.05 | 13.63 | 5.35 | 13.63 | 86.81 | 0.997 | 548 |
| Hl\_2 | 642.57 | 13.51 | 5.31 | 13.51 | 69.01 | 0.997 | 452 |
| Hl\_3 | 820.09 | 16.70 | 5.80 | 16.70 | 81.81 | 0.997 | 531 |
| Hl\_5 | 820.00 | 27.82 | 6.24 | 27.82 | 83.10 | 0.997 | 538 |
| Hl\_6 | 762.45 | 44.90 | 6.45 | 44.90 | 87.39 | 0.997 | 554 |
| Hl\_11 | 602.06 | 11.27 | 5.15 | 11.27 | 67.90 | 0.998 | 452 |
| Pp\_1 | 681.78 | 27.26 | 5.86 | 27.26 | 78.34 | 0.997 | 491 |
| Pp\_2 | 611.03 | 17.48 | 5.41 | 17.48 | 71.49 | 0.997 | 460 |
| Pp\_3 | 625.36 | 21.04 | 5.85 | 21.04 | 79.62 | 0.997 | 496 |
| Pp\_4 | 651.48 | 12.47 | 5.39 | 12.47 | 72.00 | 0.997 | 472 |
| Pp\_5 | 471.04 | 23.73 | 5.91 | 23.73 | 57.03 | 0.998 | 379 |
| Ss\_1 | 709.60 | 19.03 | 5.82 | 19.03 | 82.43 | 0.997 | 538 |
| Ss\_2 | 680.75 | 26.58 | 5.96 | 26.58 | 75.17 | 0.998 | 517 |
| Ss\_3 | 690.61 | 27.88 | 6.01 | 27.88 | 80.21 | 0.997 | 513 |
| Ss\_4 | 825.04 | 49.22 | 6.54 | 49.22 | 89.66 | 0.996 | 554 |
| Ss\_5 | 661.49 | 45.36 | 6.36 | 45.36 | 80.88 | 0.997 | 511 |
